# Determining the effects of exercise after smoking cessation therapy completion on continuous abstinence from smoking: Japanese study protocol

**DOI:** 10.1186/s13063-019-3820-7

**Published:** 2019-12-16

**Authors:** Yuka Ozaki, Maki Komiyama, Kenji Ueshima, Hiroyasu Iso, Satoko Sakata, Ayumi Morino, Mitsuyoshi Takahara, Satoshi Noguchi, Yoshihiro Kuwabara, Yuko Takahashi, Koji Hasegawa

**Affiliations:** 1 Clinical Research Institute, National Hospital Organization Kyoto Medical Center, 1-1 Mukaihata-cho, Fukakusa Fushimi-ku, Kyoto, 612-8555 Japan; 2 Center for Accessing Early Promising Treatment, Kyoto University Hospital, Kyoto, Japan; 3 Public Health Graduate School of Medicine Osaka University, Osaka, Japan; 4 Department of Epidemiology and Public Health, Graduate School of Medical Sciences, Kyushu University, Fukuoka, Japan; 5 Department of Clinical Nursing, Shiga University of Medical Science, Citsu, Shiga Japan; 6 Department of Metabolic Medicine, Osaka University Graduate School of Medicine, Osaka, Japan; 7 Section of Geriatric Dentistry Department of General Dentistry Fukuoka Dental College, Fukuoka, Japan

**Keywords:** Smoking cessation, Exercise, Weight gain, Obesity

## Abstract

**Background:**

Despite a steady world-wide decline over recent decades, rates of smoking remain high in developed countries. In Japan, 30% of men and 10% of women are smokers. Based on these rates, 18.8 million (14.06 million men and 4.74 million women) in Japan are smokers. The rate of success for smoking cessation has recently improved due to the widespread availability of drug therapy; however, the success rate for quitting smoking one year after beginning therapy is only around 50%. Previous studies have demonstrated that exercise can relieve mental stress during continuous abstinence from smoking and curb smoking resumption. To date, no large-scale, randomized controlled trials have examined the effects of exercise on smoking cessation. The present study aims to determine the effects of exercise instruction on continuous abstinence from smoking after completion of smoking cessation therapy.

**Methods:**

This is a multicenter, prospective, parallel-group, randomized controlled trial in Japan. We will enroll 300 individuals visiting a smoking cessation clinic (over 3 months) who have abstained from smoking in the second month after their initial visit as potential participants. Participants will not habitually exercise and will need to consent to participate. Participants will be randomly assigned to the exercise intervention group or control group. The intervention group will receive instruction on exercises that can be incorporated into their daily lives. The control group will be followed during the standard smoking cessation support program. The primary endpoint will be the continuous abstinence rate, and secondary endpoints will be weight, blood pressure, exhaled carbon monoxide concentration, psychological state, and blood test results. These indices will be compared between the intervention and control groups, with follow-up periods of 9 months in both groups.

**Discussion:**

By examining the effects of exercise instruction after completion of 12-week smoking cessation therapy, this study should yield quality information that can be used to develop protocols to improve the continuous abstinence rate and inhibit weight gain after smoking cessation therapy.

**Trial registration:**

UMIN Clinical Trials Registry, UMIN000014615. Registered on 1 October 2014.

## Background

### Smoking is a major health issue in Japan

Smoking is a major health issue that is associated with the development of malignancies, respiratory diseases, and cardiovascular diseases such as cerebrovascular disease and ischemic heart disease. The Japan Collaborative Cohort Study of 95,000 Japanese participants indicated that smoking increases the risk of death due to cardiovascular disease 1.6–2.0-fold. Moreover, numerous Japanese cohort studies have reported that the population attributable risk of cardiovascular disease due to smoking is approximately 20% for men and > 5% for women [1]. In Japan, the current rate of smoking is 30% in men and 10% in women. Despite a steady decline over the last few decades, smoking rates remain high in developed countries [2]. In Japan, > 1 million people suffer from smoking-related conditions such as malignancies, cerebrovascular diseases, or cardiovascular diseases, and the medical expenses attributed to smoking (and passive smoking) are estimated to be as high as 1.49 trillion Yen [3].

### Quitting smoking promotes public health

Quitting smoking has numerous preventive effects, such as an improved prognosis for avoidance of cardiovascular disease [4]. Furthermore, symptoms of chronic bronchitis improve 1–2 months after smoking cessation; patients with mild-to-moderate chronic obstructive pulmonary disease have improved pulmonary function 1 year after quitting smoking; risk of coronary artery disease decreases within 2–4 years; patients with a history of coronary artery disease have a 35% lower risk of recurrence or death; and former smokers have a rate of diminished pulmonary function equivalent to that of nonsmokers after 5 years. Moreover, individuals aged 40–59 years who attempt to quit smoking have a significantly lower risk of incurring massive medical expenses in the future compared with continuing smokers, and individuals attempting to quit smoking have a reduced risk of massive medical expenses similar to that of lifetime nonsmokers [5]. Thus, actively promoting smoking cessation should significantly inhibit the development of cardiovascular disease and greatly reduce medical expenses.

### Smoking cessation aids lack sufficient long-term effectiveness

Many people smoke again within 1 year after the initial consultation at the smoking cessation clinic, even if they quit smoking successfully. However, if they could continue quitting smoking for more than 1 year, fewer people would smoke again. Since the term of the smoking cessation therapy at clinics in Japan is 3 months, it is necessary to follow up for the remaining 9 months after the 3-month therapy (total of 1 year). In April 2006, Japanese national health insurance started to include smoking cessation treatment, and covers treatment for 3 months. The rate of success for individuals quitting smoking has recently improved due to the widespread availability of drug therapy at smoking cessation clinics. Since its introduction to the Japanese market in 2008, varenicline tartrate, an oral smoking cessation aid, has increased the success rate of smoking cessation treatment immediately after completion to approximately 70%. However, the continuous abstinence rate drops to approximately 50% 1 year after start of therapy [6]. This highlights the urgent requirement for medical personnel to provide support to individuals attempting to quit smoking to help them avoid resuming the habit.

### Exercise can improve the rate of quitting smoking

Exercise can relieve mental stress, during continuous abstinence from smoking, and curb smoking resumption [7]. A systematic review of the efficacy of exercise showed that it helped to curb smoking resumption in 12 out of 14 studies [8]. Similarly, a meta-analysis also indicated that exercise, as part of a cardiac rehabilitation program, significantly reduced the smoking rate [9]. Active incorporation of exercise may improve the rate of quitting smoking.

### Exercise can inhibit weight gain after quitting smoking

Weight gain typically occurs after smoking cessation. A recent meta-analysis reported that people on average gain 4.7 kg in weight 1 year after quitting smoking [10]. Weight gain after smoking cessation may lead to the resumption of smoking, and preventing weight gain is a vital aspect of smoking cessation support. Japanese smoking cessation programs using drug therapy provide instruction primarily in the form of diet and cognitive behavioral therapy; however, several studies have reported that such programs may not result in adequate weight control [11, 12]. Explaining specific forms of exercise to individuals who are attempting to quit smoking and do not exercise may help to improve the success rate for quitting smoking and help manage subsequent weight control.

Several intervention studies have examined the effects of combining exercise instruction with drug therapy. In a systematic review of 20 studies (including 5870 participants) examining the effectiveness of exercise interventions, the largest of the 20 studies was an Internet trial of 2318 individuals. Of the 20 studies, 8 had fewer than 30 subjects, 9 included only women, and 1 included only men. Although 4 of 20 studies reported adequate sample size and the effectiveness of exercise in smoking cessation, the effect of exercise instruction on smoking cessation remains unclear [13].

## Study objective

The present study aims to determine the effects on continuous abstinence from smoking of exercise instruction provided after completion of 12-week smoking cessation therapy (covered by the national health insurance).

## Methods/design

### Study design

This will be a multicenter intervention study with centralized enrollment (open-label, prospective, parallel-group, randomized controlled trial). Three institutions participated in this study (Kyoto Medical Center, Kumamoto Red Cross Hospital, and Komoro Kosei Hospital). Two of the facilities are located on the west side of Japan and one on the east side.

### Sample size

Previous research has shown that the addition of exercise instruction increased the odds ratio of successful smoking cessation threefold after 6 months. Of the 306 people screened by phone in this study, 40 people were considered eligible and gave consent to participate. After the 2-week run-in period, 26 individuals were randomized to the intervention and control groups, including 13 women and 12 men (excluding 1 participant who developed lung cancer) [14]. Assuming that the addition of exercise instruction after the anticipated conclusion of drug therapy is estimated to increase the odds ratio twofold after 9 months, and the standard therapy group is estimated to successfully quit smoking at a rate of 50%, then for power of 1−β = 0.8 and type I error of α = 0.05 the minimum sample size required would be 134 participants per group, making a total of 268 participants. Assuming a dropout rate of 10% during the study, a randomized intervention study with 150 participants per group will require a total of 300 participants [15]. Study participants will be allocated to one of two groups: an exercise intervention group, who will receive additional exercise instruction in addition to the standard instruction, and a control group, who will receive the standard instruction. Participants will be followed for 9 months after enrollment. The target number of participants to be enrolled is 300 (150 in each group).

### Participants

Study participants will include individuals who have abstained from smoking during the last month while undergoing standard smoking cessation therapy (3 months after the initial visit).

### Inclusion criteria

Individuals who have abstained from smoking during the last month while undergoing standard smoking cessation therapy (3 months after the initial visit) and fulfilling the following will be included: (1) individuals who do not exercise (those who do not answer “I have continued to exercise 30 min a day at least twice a week for over a year” in a questionnaire); (2) age 20–75 years; and (3) individuals who agree to the purpose of this study and who provide written consent.

### Exclusion criteria

Individuals fulfilling any of the following items will be excluded: (1) those prohibited from exercising by their physician (individuals for whom exercise is contraindicated); (2) those who would have difficulty exercising due to conditions, such as an orthopedic disorder, neuromuscular disease, or peripheral vascular disease; (3) pregnant women; (4) in-patients or residents of a facility; or (5) individuals whose participation in this study has been deemed inappropriate by their primary physician.

### Discontinuation criteria

This study will be discontinued in the event of any of the following: (1) continuation of the study is not feasible due to adverse events; (2) the study cannot be continued due to participant withdrawal or withdrawal of consent; (3) participants meet the exclusion criteria or are deemed ineligible after enrollment; (4) women who are pregnant; (5) marked noncompliance; (6) the study itself is discontinued; or (7) an investigator otherwise deems that continuing this study is not feasible.

### Ethical considerations

All procedures will be carried out in accordance with the ethical standards of the facilities involved and those of domestic research councils and in accordance with the 1964 Helsinki Declaration and its subsequent amendments or comparable ethical standards.

### Study protocol

**Fig. 1:**
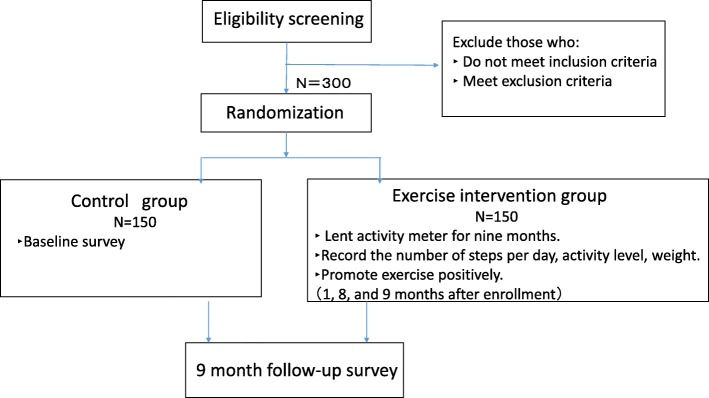
Overview of study protocol

**Fig. 2:**
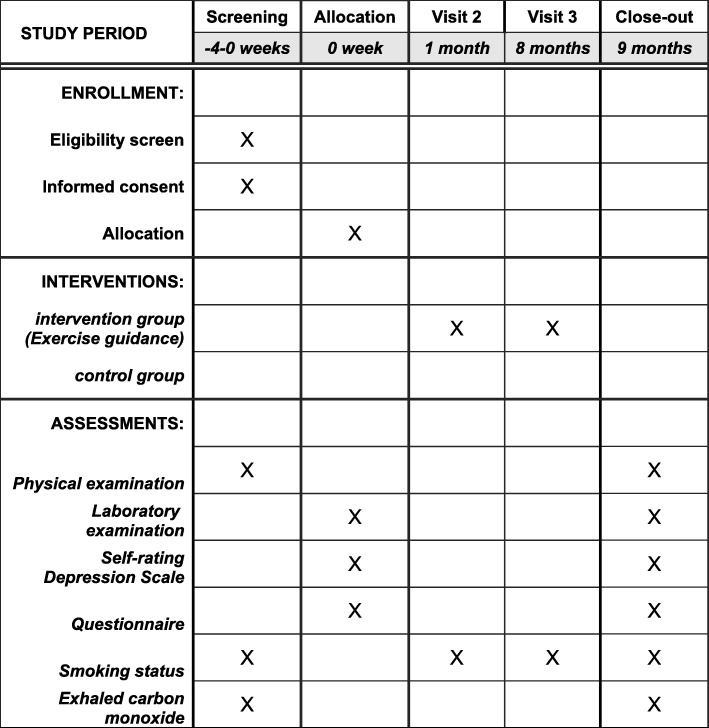
Schedule of enrollment, interventions, and assessments

An overview of the proposed study protocol is shown in Fig. 1. The schedule of enrollment, interventions, and assessments is shown in Fig. 2. The Standard Protocol Items: Recommendations for Interventional Trials (SPIRIT) Checklist is provided in Additional file 1.

### Allocation

Participants will be centrally allocated using the electronic data capture (EDC) system of the University Hospital Medical Information Network. After written consent is obtained from participants who meet the selection criteria, the lead investigator will verify that the participants meet all of the eligibility criteria and none of the exclusion criteria, and their information will be registered in the EDC system. After registration, the EDC system will assign patient numbers that do not include a patient’s personal information. Immediately after entry, the EDC system will randomly assign participants to one of two groups (exercise intervention or control groups) by dynamic allocation.

### Allocation and allocation adjustment factors

Potential confounding factors (age, sex, number of cigarettes smoked per day, Fagerstrom test for nicotine dependence (FTND) score, and self-rating depression scale (SDS) score) will be adjusted between the two groups via registration in the EDC system. Participants will be randomized by minimization, which is a method of dynamic allocation.

### Intervention

#### Acquisition of data at the beginning of the study

Patient characteristics (sex, age, medical history, history of present illness, alcohol consumption, type of medications taken, and psychological state), blood chemistry measurements (white blood cell count, red blood cell count, hemoglobin, hematocrit, platelet count, fasting blood glucose, total cholesterol (T-cho), high-density lipoprotein cholesterol (HDL-cho), and triglyceride (TG)), and measurements of height, weight, blood pressure, and concentration of exhaled carbon monoxide will be examined at the beginning of the study. Smoking status (number of years of smoking, number of cigarettes smoked, tobacco dependence screener score, FTND score, age the individual started smoking, smokers in the family, and number of previous attempts to quit smoking) will be recorded during an interview at the initial visit to the smoking cessation clinic.

#### Exercise intervention group

After completion of the smoking cessation therapy, participants will be informed of the significance of active exercise while attempting to quit smoking. Participants will receive an activity tracker for the duration of the study and will be instructed on exercises to increase and maintain their physical activity as part of their daily lives.

#### Setting of exercise goals

During follow up, participants will individually record their daily step count, level of activity, and weight. Participants will be regularly followed up approximately 2–4 times over 9 months. During regular follow up, participants will receive feedback based on their individual logs, their exercise goals will be adjusted, and they will be encouraged to maintain their amount of physical activity and control their weight. Exercise instruction will be provided in accordance with an exercise instruction manual compiled under the supervision of a certified fitness instructor and a certified cardiac rehabilitation specialist.

#### Standard therapy (control) group

The control group will be followed during the course of the standard smoking cessation program. After completing smoking cessation therapy, participants will not be actively advised to exercise; therefore, further exercise instruction will not be provided.

Participants will receive an activity tracker immediately after completing smoking cessation therapy and 8 months after beginning the study. They will return the tracker 1 month later in both instances. Participants will be regularly followed up 2–4 times over 9 months. During regular follow up, we will confirm whether smoking cessation has continued, and participants will not receive active instructions for exercise.

#### Data collected during the study and follow-up periods

The study will begin on the day the participant is given an activity tracker. The follow-up period will be 9 months in both groups. In addition to face-to-face meetings, follow up may be conducted by mail, telephone, or on line. Participant status will be ascertained at the beginning of the study and at 1, 8, and 9 months after beginning the study. Continuous abstinence from smoking, psychological state, type of medications taken, blood chemistry measurements (white blood cell count, red blood cell count, hemoglobin, hematocrit, platelet count, fasting blood glucose, and T-cho, HDL-cho, and TG), and measurements of height, weight, blood pressure, and concentration of exhaled carbon monoxide) will be assessed in both groups after 9 months.

#### Items observed and studied

Primary endpoint: the primary endpoint will be continuous abstinence rate. An individual is deemed to have continuously abstained from smoking if they report when interviewed that they have not smoked in the past week and if their concentration of exhaled carbon monoxide is ≤ 7 ppm [12].

Secondary endpoints: the secondary endpoints are changes in metabolic indices (height, weight, blood pressure, exhaled carbon monoxide concentration, red blood cell count, white blood cell count, hemoglobin, hematocrit, platelet count, fasting blood glucose, T-cho, HDL-cho, and TG) and changes in an individual’s psychological state (SDS score, depressive tendencies, and patient activation measure score).

### Analysis

#### Analysis set

The two groups (exercise intervention and control groups) will be compared based on the intention-to-treat principle. All data for the metabolic indices and psychological states of the participants in each group obtained upon enrollment and 9 months after enrollment will be analyzed. Individuals who quit smoking for a prolonged period will be similarly analyzed and those who resume smoking will be excluded. Quitting smoking for a prolonged period is defined as not smoking for 9 months after enrolling in the study (determined by interview) and having exhaled carbon monoxide levels ≤ 7 ppm. The continuous abstinence rate is defined as the proportion of participants who abstain from smoking with respect to the total number of participants in each group.

#### Items analyzed and analytical methods

The characteristics of the participants in both groups will be described statistically. Changes in the metabolic index and psychological state from enrollment to 9 months after enrollment will be compared between the two groups. The distribution of individual indices at baseline and 9 months after enrollment will be determined in the two groups, and the *t* test or a Wilcoxon rank-sum test will be performed. Analysis of covariance, multiple regression, or logistic regression will be used for factor adjustment. In addition, Fisher’s exact test will be used to compare the continuous abstinence rate in the two groups. The level of significance will be *p* ≤ 0.05. Two-tailed alpha of 5% and two-sided 95% confidence level will be used. A statistician will perform the statistical analysis.

#### Early termination of this study

The trial will be discontinued in the event of any of the following situations: (1) if the trial cannot be safely conducted due to serious adverse events; (2) if it is not possible to enroll the planned sample size; or (3) if it is not feasible to continue the study.

## Discussion

Exercise has various effects, including reduction in the risk of osteoporosis due to improvements in cardiovascular, musculoskeletal, and pulmonary function, and increased muscle mass, and prevention of obesity due to fat reduction and increased muscle mass, prevention of dyslipidemia due to reduced serum TG and increased HDL-cho, prevention of diabetes mellitus due to alleviation of insulin resistance, and prevention of hypertension due to improved vascular endothelial function and reduced blood pressure [16, 17]. Exercise also relieves stress and is an effective treatment for depression as it alleviates anxiety [18]. The 2013 Physical Activity Guidelines to Promote Health were formulated in Japan in 2013 [19] and included exercise as an important component.

Weight gain is common after quitting smoking [20] and impairs glucose tolerance [21–23], and may lead to smoking resumption [24, 25]. In this study, simple and safe exercises that can be easily performed by individuals who have gained weight after quitting smoking will be performed in accordance with a cardiac rehabilitation program. Participants will record their weight and step count, which should increase their self-efficacy. This study combines smoking cessation therapy and exercise therapy. If the study shows that exercise instruction improves the continuous abstinence rate, widespread implementation of the intervention is anticipated to reduce the public smoking rate, help to promote health, reduce medical expenses, and greatly benefit the public.

### Trial status

This study protocol is the first version since 10 December 2015. Recruitment began on 30 March 2016, and the expected recruitment completion date is December 2019. Recruitment to this study is ongoing at the time of manuscript submission.

## Supplementary information

**Additional file 1:** SPIRIT 2013 Checklist: Recommended items to address in a clinical trial protocol and related documents*.

## Data Availability

Not applicable.

## Abbreviations

EDC: Electronic data capture
FTND: Fagerstrom test for nicotine dependence
HDL-cho: High-density lipoprotein cholesterol
SDS: Self-rating depression scale
T-cho: Total cholesterol
TG: Triglyceride
